# Nurturing Acceptance for Research in the Community: Conceptualising Engagement Towards Research Through Normalisation Process Theory

**DOI:** 10.1111/hex.70356

**Published:** 2025-08-17

**Authors:** Tanvir C. Turin, Nashit Chowdhury, Tanmoy Newaz, Mohammad M. H. Raihan, Nafiza Rahman, Nahid Rumana

**Affiliations:** ^1^ Department of Family Medicine, Cumming School of Medicine University of Calgary Calgary Alberta Canada; ^2^ Department of Community Health Sciences, Cumming School of Medicine University of Calgary Calgary Alberta Canada; ^3^ Community Scholar and Citizen Researcher Calgary Alberta Canada

**Keywords:** co‐creation, co‐design, community engagement, meta research, transdisciplinary

## Abstract

**Background:**

Community‐engaged research with immigrant and visible minority communities requires intentional strategies to foster acceptance, trust and sustained participation. Historically, research in marginalised communities has been extractive and externally driven, leading to mistrust and scepticism.

**Objectives:**

To address this, we applied Normalisation Process Theory (NPT) as a guiding framework to integrate research as a meaningful, community‐driven practice rather than an extractive academic exercise. The objective of this paper is to describe how NPT can illuminate the social and relational processes involved in introducing, legitimising and maintaining collaborative research practices within a community.

**Methods:**

Using NPT's four constructs—Coherence (establishing the ‘why’), Cognitive Participation (generating the ‘will’), Collective Action (carrying out the ‘tasks’) and Reflexive Monitoring (reflecting and adapting)—we structured a phased approach to community engagement. Our initiatives included community‐focused outreach, community organisation for capacity‐building and collaborative research activities, all designed to shift research from being externally imposed to community‐engaged. A key challenge was achieving initial acceptance of research within the community.

**Results:**

Through intentional outreach, inclusive recruitment and participatory knowledge production, we transitioned from establishing legitimacy to building long‐term, community‐driven partnerships.

**Conclusion:**

Our experience highlight the importance of embedding research within everyday community life, valuing local expertise and ensuring that knowledge production remains collaborative, accessible and action‐oriented. This approach not only bridges the gap between academia and the community but also fosters equitable, enduring research relationships that lead to meaningful, sustainable impact.

**Patient or Public Contribution:**

While preparing this manuscript, we have partnered actively with community scholars and citizen researchers from the very beginning. We had regular interactions with them to get their valuable and insightful inputs in shaping our reflections. Their involvement as co‐authors in this paper also provided a learning opportunity for them and facilitated them to gain insight into knowledge engagement. All authors support the greater community/citizen/public involvement in research in an equitable manner.

## 1 Introduction

### 1.1 Community‐Engaged Research

Community‐engaged research is a broader framework and multifaceted approach to research that encompasses various levels of community involvement and collaboration between academic researchers and community members or organisations [1]. Inclusive participation ensures that unique insights are captured, enhances understanding of the topic and produces results that accurately reflect community members' lived experiences, leading to practical applications. Community‐engaged research is especially vital for understanding the perspectives of marginalised groups, such as immigrants, and for co‐developing effective, culturally relevant solutions that address their unique challenges. This approach facilitates authentic partnerships among individuals who are affiliated with or self‐identify based on geographic proximity, shared interests or comparable circumstances. Community‐engaged research also emphasises the integration of community perspectives and expertise throughout the research process, thereby enhancing the relevance and potential impact of the study outcomes [1]. This is achieved through partnership and co‐learning, with community members contributing their knowledge and expertise to shape the research agenda, design and implementation [2]. This requires involvement of community members in various stages of the research process beyond tokenistic participation [3]. By leveraging the unique and complementary experience and expertise of both researchers and community members, community‐engaged research allows for the initiation of community‐informed research and work towards outcomes that are more applicable to the complex needs and priorities of the community [1].

### 1.2 Community Engaged Programme of Research With Immigrant/Visible‐Minority Community

Our transdisciplinary programme of research, initiated in 2014 and housed within the Cumming School of Medicine at the University of Calgary, is committed to advancing the wellness of the immigrant/visible‐minority communities. We strive to address health and social disparities, systemic and structural barriers to integration, and inequities in health and social care access by working in close partnership with the community we belong to, live in and conduct research in. Our interdisciplinary, cross‐sectoral research team bridges academia, the public and private sectors, and the community. As university‐based academics, we leverage research methodological expertise, institutional support and networking opportunities spanning public, private and civic sectors. We bring together academic researchers, learners at all levels, community leaders, local organisations and government partners, collaborating closely at every stage of the research process. Guided by community‐engaged research principles, we aim to ensure that our studies are not only academically meaningful but also practically valuable and culturally relevant. As a first initiative, we started working with a visible‐minority community (the Bangladeshi‐Canadian community) in Calgary [4, 5, 6, 7]. Our ongoing efforts to meaningful community engagement (CE) [8] were rooted in inclusive, ethical, democratised and decolonised research practices, which entail ensuring diverse voices are valued and represented, maintaining integrity and respect for participants, and challenging colonial biases while promoting community‐led knowledge systems [3, 9]. By centring our efforts ‘in the community, with the community, and for the community’, we strive to move beyond traditional academic boundaries and cultivate a participatory, impactful and empowering model of research—one that is guided by the values, needs and aspirations of the communities we work alongside. We immerse ourselves in the community by conducting research in accessible, culturally relevant spaces, such as local cultural entities, religious centres, businesses, and community and grassroots organisations [9, 10].

This approach demonstrates our commitment to understanding the unique unmet needs, systemic barriers and lived experiences of the community while rejecting extractive and exploitative research models, which prioritise academic or institutional gains over community benefit and often disregard community autonomy and well‐being. Instead, we prioritise co‐learning and reciprocal knowledge exchange, integrating community perspectives into every phase of the research process. Through this, we hope to move away from extractive research models and move towards approaches that honour and amplify community voices.

### 1.3 The Challenge: From Scepticism to Partnership—Earning Community Acceptance and Fostering Research Trust

Through our initial CE efforts for our programme of research, we quickly realised a significant impediment to meaningful research engagement. A common assumption in CE for research is that community members will innately regard research and researchers as favourable once they comprehend the project's overall purpose and potential benefits. Yet, our experience diverged from this expectation. Despite our efforts to explain how the programme of research could serve the community's interests, we observed an undercurrent of scepticism and reluctance. Several factors appeared to contribute to this hesitation. First, many community members were not accustomed to academic research activities and thus found it difficult to understand how or why researchers from external institutions wanted to study them. This unfamiliarity frequently manifested as questions about what research entails, why academics were so interested in their particular experiences, and how the findings would ultimately be used. The broader lack of research as a regular facet of life meant that, when approached by academics, community members often did not have a clear framework for deciding whether, how or why to participate. Second, historical contexts of colonialism and exploitative research practices have left enduring impressions that persist and continue to inform how communities perceive academic inquiries. Many communities—particularly marginalised groups—have inherited a collective memory of research projects that were extractive rather than collaborative, ultimately benefiting the researchers in their careers or achieving other agendas rather than bringing some tangible outcomes for the participants. This legacy casts a shadow of doubt on new research initiatives, even if they aim to operate ethically and inclusively. Consequently, our team encountered comments or concerns suggesting apprehension about sharing personal experiences or contributing data because the research process and potential outcomes were not fully understood.

These observations highlighted the pivotal importance of creating acceptance of both the research and the researchers themselves as an essential precursor to building trust. We learned that while *acceptance* generally reflects a basic willingness to allow research activities, *trust* signifies a deeper conviction that the research will be conducted ethically, transparently and with genuine intention for the community's well‐being. In our work with the Bangladeshi‐Canadian community in Calgary, our research focused on understanding barriers to health and social service access, identifying unmet needs and co‐developing culturally relevant solutions to improve community well‐being. In practical terms, this involves investing significant time in relationship‐building activities, actively listening to community concerns, acknowledging lived experience of the community and valuing their expertise and perspectives. To ensure our engagement was meaningful and accessible, we held all our activities in spaces familiar and comfortable to the community, such as cultural festivals, ethnic restaurants and religious spaces like mosques. Rather than quickly launching into data collection, we recognised the need to prioritise dialogues and collaborations that align with the community's values, address their immediate concerns and establish our credibility. Through open conversations in these culturally relevant settings, we began taking steps to address uncertainty and scepticism. This meant clarifying the specific aims of our study, for example, exploring challenges in accessing healthcare or social supports, and identifying strengths within the community. We encouraged community members to become actively involved in the research, explained how the data would be used responsibly and clearly outlined the benefits for the community—such as improved health literacy, enhanced service access, community‐led initiatives or more responsive local policies. Additionally, we recognised that trust is not a one‐time accomplishment but an ongoing process, maintained by consistent and transparent engagement, regular bidirectional feedback, and a commitment to co‐create knowledge rather than simply extracting it. In sum, the initial resistance we faced underscores a larger truth about CE: establishing rapport and legitimacy within the community is not automatic but must be cultivated through sustained engagement, cultural humility and ethical sensitivity. Only by addressing doubts rooted in both present‐day realities and historical precedents can we foster an environment in which genuine collaboration—and, ultimately, meaningful research outcomes—can flourish. Meaningful and continuous CE stands as a pivotal precursor to building a robust programme of research in the community, requiring dedicated outreach and organising that first fosters acceptance, and then deepens into trust towards the value of research.

## 2 Community Engagement to Increase Awareness and Acceptance, Leading to Collaboration and Partnership in Our Research Programme: Using the NPT

**Figure 1:**
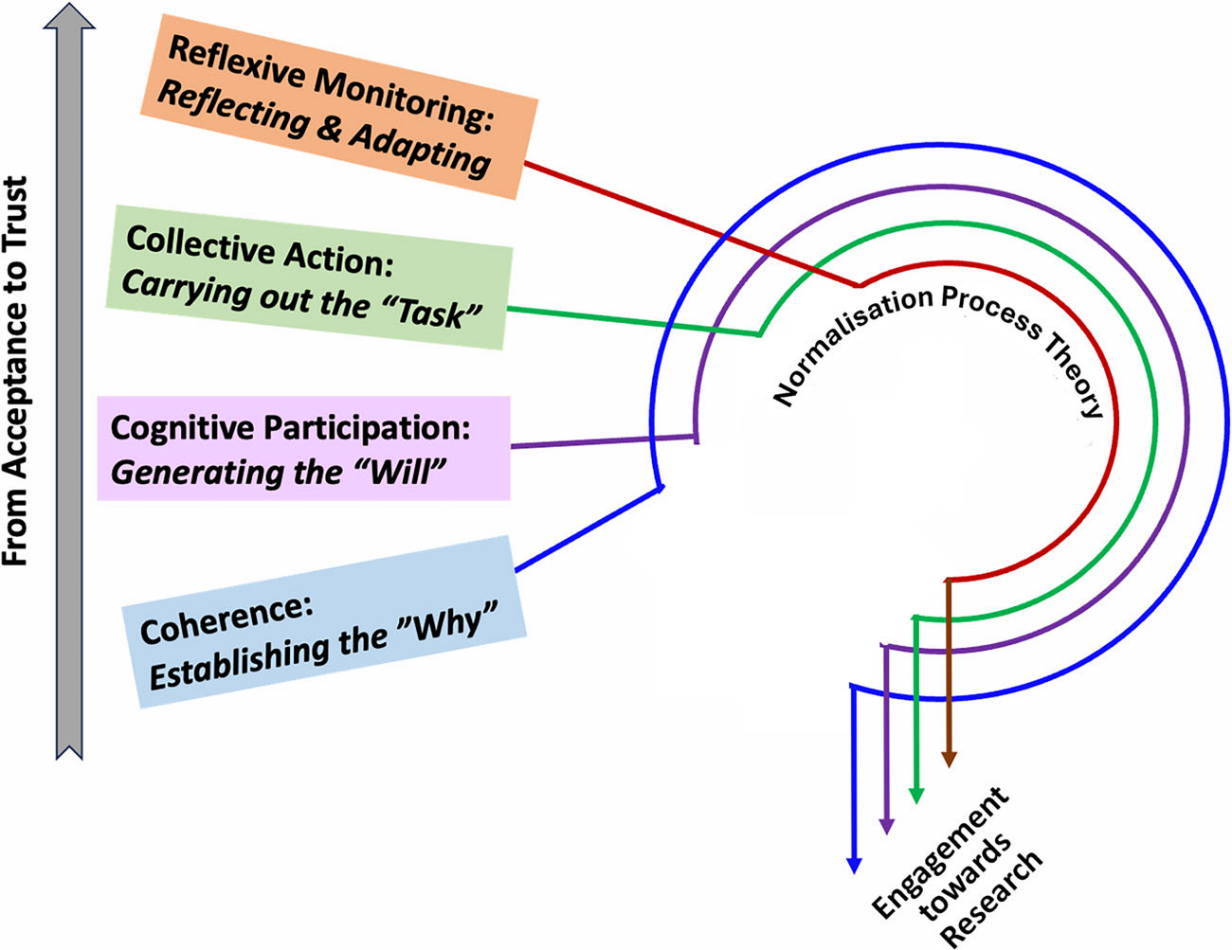
Constructs of Normalisation Process Theory.

Establishing an effective CE strategy is essential for any research endeavour that aims to gain acceptance and foster genuine collaboration and trust. In our case, engaging with the Bangladeshi community in Calgary required not only raising awareness about the value and relevance of our research programme, but also addressing scepticism born from historical experiences of extractive or tokenistic research. By placing emphasis on open dialogue, cultural relevance and tangible community benefits, we aimed to create an environment in which the community would accept our research, and its members would feel empowered to participate as equal partners rather than mere participants of the study. To structure and guide our engagement activities, we strategically adopted the tenets of Normalisation Process Theory (NPT) [11, 12]. NPT explains how new practices, interventions or ways of working become routinely embedded (‘normalized’) in social or community contexts. It is grounded in the assumption that implementation and sustainability of new practices are fundamentally social processes, shaped by collective action, shared understanding and ongoing appraisal among stakeholders. NPT's focus on the social mechanisms of embedding new practices aligns closely with the challenges of fostering acceptance and trust in community‐engaged research. As illustrated in Figure 1, NPT consists of four main constructs on (a) coherence (how people understand and make sense of a new practice), (b) cognitive participation (how people engage and commit to it), (c) collective action (how people work together to enact it), and (d) reflexive monitoring (how people evaluate and sustain it). These constructs provide a structured approach for the iterative, relational work required to gain acceptance and foster trust during CE. In our context of CE aimed at earning acceptance and fostering trust for a research programme, NPT has been a useful framework because it highlights the social and relational processes involved in introducing, legitimising and maintaining new idea and practice—in this case, collaborative research endeavours. Rather than adopting NPT as a rigid, overarching framework a priori, we iteratively engaged with its core tenets throughout the development of each community‐engaged initiative and then conducted a post hoc analysis to construe our observations against those same constructs. The following sections detail how we applied NPT to increase community awareness, acceptance and eventual collaboration in our research programme.

### 2.1 Coherence

Coherence, as defined in the context of NPT, signifies the extent to which individuals comprehend and make sense of a new practice or initiative, along with its purpose, benefits and requirements [12]. In our CE efforts within the Bangladeshi‐Canadian community, we prioritised this NPT component at the outset, aiming to establish CE as a normalised and routine aspect. Recognising the non‐linear and dynamic relationships between different elements of the implementation/adoption process, our overarching objective was to cultivate a norm of acceptance towards research activities within the community. To address coherence in our CE efforts, we took proactive steps to ensure that the community not only became aware of our programme of research but also understood and got involved in our research endeavours. Our research team, benefiting from having members of the Bangladeshi‐Canadian community, could readily attain a comprehensive understanding of the community ecosystem—encompassing its human, social and cultural characteristics. This advantage enabled us to identify and engage with influential community figures, including community champions representing various segments—men, women, youth and subgroups distinguished by religion, profession or regional affiliations within Bangladesh. Through these champions, we gained insights into the community's beliefs and attitudes regarding CE and how best to foster coherence throughout the broader community. The community champions acted as liaisons, helping members differentiate the new CE and research practices, relate them to their personal goals and values, and appreciate the significance of our initiatives within their cultural contexts. As we clarified our approach and research goals, the champions vetted our efforts and relayed our message to the wider community in accessible language. Consequently, community members were able to understand our purpose, potential benefits and the actions required for their possible involvement. We emphasised that we were seeking their guidance and support to leverage our research tools and knowledge for the community's benefit. Continuous engagement through a series of discussions with community champions, we began the active collaboration process more effectively within the community at large. This strategic approach to coherence‐building contributed significantly to establishing a foundation of understanding and sense‐making, aligning our research practices with the values and needs of the Bangladeshi‐Canadian community.

### 2.2 Cognitive Participation

Cognitive participation, within the NPT framework, refers to the relational work needed to build and sustain a new practice; in this case, participating in guiding and developing the research efforts [12]. This construct comprises four integral components: initiation, enrolment, legitimation and activation. Building upon the foundations of coherence, cognitive participation focuses on the process of getting individuals and organisations within the community to actively engage with our programme of research. In essence, cognitive participation in our programme of research entailed bringing community members together, consulting with them to understand best practices and ensuring sustained engagement, both in the present and future. This relational work was integral to building and sustaining an attitude of acceptance around our research initiatives within the Bangladeshi‐Canadian community.

In the initiation phase, we first worked alongside community champions to outline the steps we could take to engage and assess the community. We realised that we need to identify certain committed individuals who would consistently support us and help connect with the wider community. These individuals included both those formally integrated into our research team as compensated members and those who voluntarily contributed their time and efforts. To foster these connections, we engaged in regular interactions through participation in community programmes, informal gatherings and coffee meetings. These ongoing engagements allowed us to identify and build relationships with individuals who were willing to contribute at varying levels of involvement, ensuring sustained community support throughout the research process.

Enrolment, the next phase of cognitive participation, involved organising community members to collectively contribute to the work associated with CE efforts. In our context, this included exploring individual and group relationships between community members, community organisations and the research team. For example, many community members were interested in actively helping us organise certain community‐focused events, such as a workshop on Covid‐19 during the early pandemic days, and other health literacy workshops, such as those on diabetes and primary care barriers. Others showed a keen interest in being involved in our research and joining our research team in varying capacities, ranging from providing data, helping with outreach/recruitment/enrollment, collaborating in analysis/interpretation and co‐leading knowledge translation activities. As such, we planned to try to meet the needs and desires of community members to the best of our abilities to ensure that all individuals felt appreciated and heard.

With our consistent interaction, meeting and participation in community services, and arranging workshops *with* the community, we gradually become visible and recognisable within the community. This facilitated the legitimation process, and the community members started to truly believe in us that we are not there to collect data in ‘parachute in and out’ manner and leave them after our project is accomplished. At this point, the general community members really started to pay attention to us and started to believe that this way of engagement and participating in research could benefit the community at large, and it was right for them to be involved in these activities. Further, the continued support and validation from different community leaders and groups offered community members additional confidence and trust in engaging with our programme's initiatives.

Activation, the final phase of cognitive participation involved collectively defining actions and procedures needed to sustain long‐term positive attitudes towards participation in research activities. In our CE efforts, activation was evident in the ongoing commitment of community members to continue their involvement in various research activities, contributing to the success and impact of our programme. We also identified different types of community‐based organisations, including profit, non‐profit, informal organisations or groups of people, and laid out the plan for different roles they can play. As well, what capacity‐building activities would be needed to engage the participants in different roles so that they can contribute substantially to the research process. This approach is essential for sustainable and meaningful CE, especially in immigrant/ethnic‐minority communities.

### 2.3 Collective Action

Collective action, in the context of NPT, denotes the operational work required to establish and maintain a new set of practices [12]. Within our CE strategy, collective action encompassed the activities necessary to start the research process, where Bangladeshi‐Canadian community members were active research collaborators.

Within this collective action dimension, our specific initiatives focused on capacity building to encourage individuals interested in both participating in and supporting our research. This involved arranging various levels of training and mentorship based on each person's preferred level of involvement. To facilitate this, our research team, in collaboration with community members, launched three key programmes: (1) Community Scholar and Citizen Researcher (CSCR), (2) Community Think‐Tank, or Community Advisory Group, and (3) Refugee and Immigrant Self‐Empowerment (RISE), each employing a distinct focus to engage community members. The CSCR programme offers opportunities and support for scholars and citizen researchers from diverse backgrounds to join research projects, gaining experience in both academic and informal settings. The Community Think‐Tank or Community Advisory Group involves community members in interpreting research findings and co‐developing feasible, culturally acceptable solutions. This approach brings together a diverse group of community participants for co‐formulating research questions, co‐designing projects, evaluating data and disseminating findings—thus ensuring sustained community involvement throughout the research process. We also prioritised designing capacity‐building workshops that would not cease after a single project, thereby enabling interested community members to understand how their efforts benefit the broader community. Additionally, we implemented the RISE programme as part of our collective action plan to address issues identified during the coherence phase. Operating since 2016, this youth summer learning programme empowers immigrant and racialised youth to become community health and wellness champions. Through RISE, we engaged younger members in leadership development while simultaneously building trust around our CE and research activities. An essential aspect of our approach was our deliberate and ongoing effort to expand community participation in these programmes. Rather than relying on a fixed group of participants, we actively worked to broaden engagement by continuously encouraging new community members to join. We were intentional in our outreach, making a concerted effort to advertise opportunities, personally invite individuals and leverage snowball recruitment strategies to bring in fresh voices. By applying principles from the Diffusion of Innovation theory [13], we strived to extend our reach, diversify participation and foster growth in engagement within the community.

Furthermore, our commitment to collective action extended to supporting community organisations' needs. This included assisting with grant proposals for community organisations, providing career enhancement opportunities for internationally trained medical professionals, and connecting individuals or organisations with valuable resources within our network. These collective actions were instrumental in forging meaningful partnerships and establishing our reputation as a trustworthy, dependable resource in the Bangladeshi‐Canadian community.

### 2.4 Reflexive Monitoring

Reflexive Monitoring, the final construct in NPT, plays a crucial role in determining whether the practice becomes normalised or integrated into the community [12]. This process involves four interconnected components: systematisation, communal appraisal, individual appraisal and reconfiguration. To evaluate the effectiveness of our CE efforts in promoting an attitude of acceptance and trust towards community‐engaged research in the Bangladeshi‐Canadian community, we first undertook systematisation work. Specifically, we documented levels of participation and the number of co‐authored studies we published with community partners, reflecting their significant contributions that emerged from our approach. These efforts primarily focus on our work addressing barriers to primary care access among Bangladeshi communities in Canada. We also tracked participation in our CSCR, Community Think‐Tank and RISE programmes, as well as follow‐up with individuals who discontinued, to enhance the sustainability of these programmes. This strategy allowed us to gauge the impact of our CE efforts in diverse and meaningful ways. Our CE efforts also led to ongoing interactions in both formal and community settings, facilitating collaborative assessments of our initiatives. Our biannual symposium [14]—‘Mobilising Knowledge on Newcomers Symposium 2019, 2021, and 2023’—serves as a prime example of communal appraisal. To date, these three symposia have brought together community members, academic and non‐academic researchers, and community organisations to showcase community‐focused research and share implementation findings directly with community members. Participants, including those who joined our research team and organisations that collaborated on earlier projects, presented interactive group sessions, as well as oral and poster presentations. By offering this platform for reflection on both achievements and areas for improvement, we gained insightful feedback on how to refine our overall approach to serving the community. Additionally, a roundtable session at the conclusion of each symposium encouraged constructive critiques and suggestions, helping us strengthen our CE activities. The success of these symposia demonstrated that the Bengali community showed significant acceptance of and enthusiasm for future community‐engaged research projects. At the individual level, there was a need to appraise the impact of our CE practices. To enable community members to evaluate our work, we published lay articles in a local Bangla‐language newspaper summarising the events we had organised. This method of dissemination served to share research findings and empowered community members to assess the relevance and impact of our work within their personal spheres. Our inclusive approach, coupled with an open attitude, encouraged any community member to approach either our research team or community champions with feedback. This interaction significantly enhanced our connection to the broader community, thereby upholding our initial goals of transparency.

Appraisal at both the communal and individual levels allowed us to reflect on how to refine our CE strategies for greater effectiveness and sustainability. Particularly, the feedback we received from our past and transitioning participants of CSCR, RISE and Community Think‐Tank programmes helped evolve the programmes and improved our overall strategies. We aimed to ensure that our methods would yield a positive shift in attitudes towards community‐engaged research within the Bangladeshi community. By listening to feedback and suggestions from community champions and general members, we organised health‐related workshops, hosted community events and symposiums, participated in festivals, offered youth education seminars, and published event information in Bangla‐language newspapers. While this process helped us learn about the community's evolving needs, it also reinforced the trust placed in us. We remained eager to incorporate any suggestions, whether informally received at events or formally communicated through meetings with community leaders and organisations. This iterative reconfiguration approach reflected our commitment to making our CE efforts effective, adaptive and responsive to the evolving needs of the community.

## 3 Conclusion

**Figure 2:**
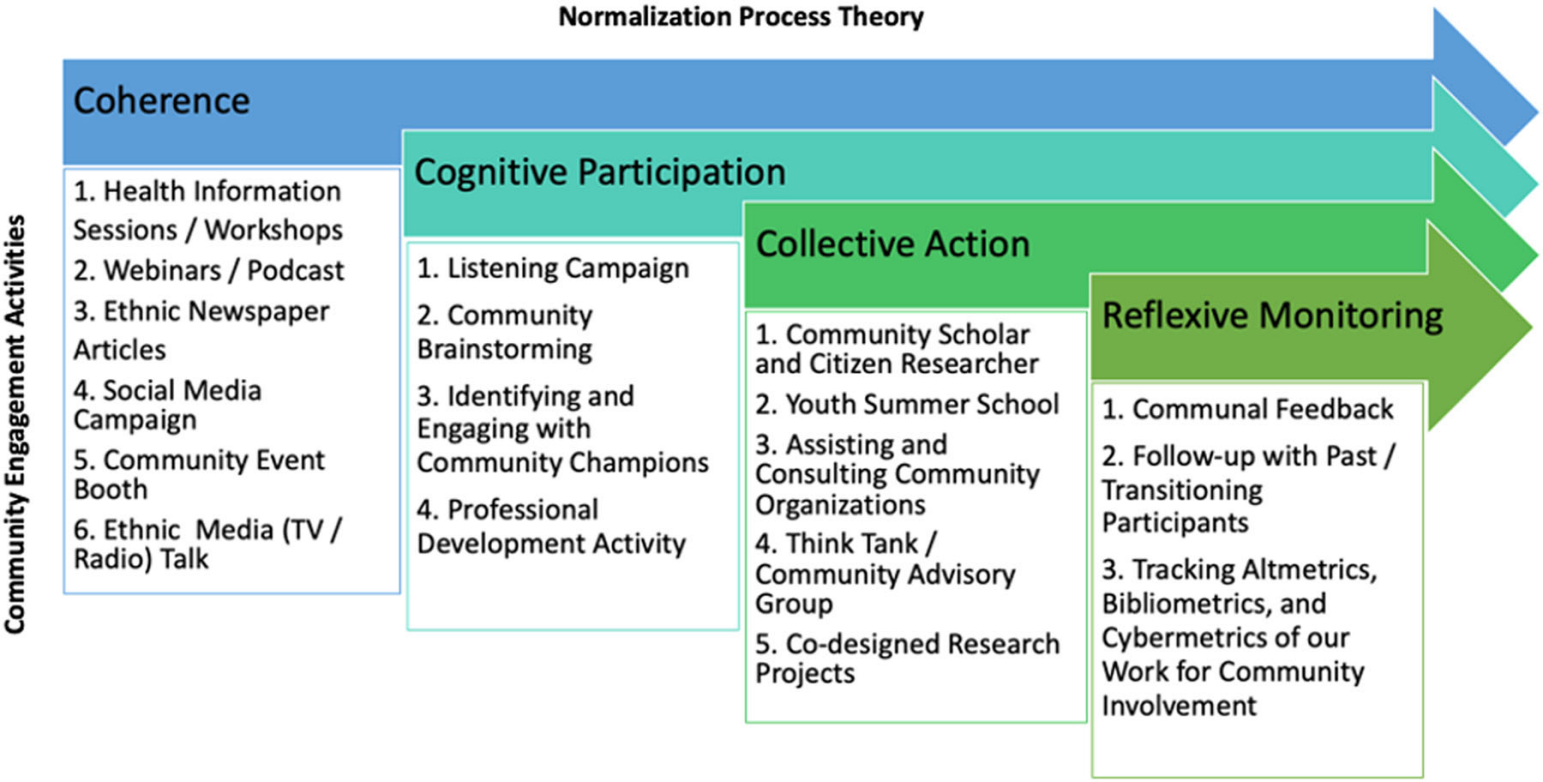
Community engagement activities guided by Normalisation Process Theory.

CE emphasises involving community members throughout the research process so that their perspectives actively shape the study. NPT provides a structured approach to embed new practices from CE into everyday community life. We employed NPT to enhance acceptance of and participation in CE efforts within the Bangladeshi‐Canadian community in Calgary, Alberta, Canada. We integrated the concepts of coherence, cognitive participation, collective action and appraisal in our ongoing CE activities with the Bangladeshi community (Figure 2). By continuously applying these principles, we moved from scepticism towards genuine partnership, underscoring that CE activities conducted amid this process strengthen community trust and advance overall research acceptance. This approach ensures that CE is not a one‐time initiative but an evolving, normalised process. As research activities become more common through NPT, communities become increasingly involved, leading to sustainable and meaningful research relationships.

## Author Contributions

T.C.T., N.Ra., and N.Ru. conceived of the paper. T.C.T., T.N., M.M.H.R., and N.C. drafted the paper. All authors provided critical input to multiple drafts. N.Ra. and N.Ru. critically reviewed the manuscript and provided important perspectives as community member researchers. T.C.T. act as guarantor to this article.

## Ethics Statement

The authors have nothing to report.

## Conflicts of Interest

The authors declare no conflicts of interest.

## Data Availability

Data sharing is not applicable to this article as no datasets were generated or analysed during the current study.
